# Effects of modified BOPPPS-based SPOC and Flipped class on 5th-year undergraduate oral histopathology learning in China during COVID-19

**DOI:** 10.1186/s12909-021-02980-6

**Published:** 2021-10-26

**Authors:** Shan Wang, Xin Xu, Fang Li, Haixia Fan, Eryang Zhao, Jie Bai

**Affiliations:** 1 Department of Oral Pathology, Hospital of Stomatology, The First Affiliated Hospital, Harbin Medical University, Harbin, 150001 P. R. China; 2 Department of Oral Medicine, Heilongjiang Nursing College, Harbin, 150001 P. R. China; 3 Department of Stomatology, Women’s and Children’s Medical Center, Haikou, 570000 P. R. China; 4 Department of Oral Medicine, Jining Medical College, Jining, 272067 P. R. China; 5 Department of Ophthalmology, The Fourth Affiliated Hospital, Zhejiang University School of Medicine, Yiwu, 322000 P. R. China

**Keywords:** BOPPPS, SPOC, Flipped class, Oral histopathology

## Abstract

**Background:**

Colleges and universities in China have offered courses based on online teaching platforms as required by the Ministry of Education since the beginning of the COVID-19 pandemic. This emergency action was not an expedient measure, but a powerful impetus to improve extant education and implement teaching reform. Oral histopathology is a basic subject in oral medicine education, which combines theory with practice. The course aims to improve the ability of students to observe, think, analyze and identify oral diseases.

**Method:**

We adjusted and modified the original Bridge-In, Outcomes, Pre-assessment, Participatory Learning, Post-assessment, and Summary (BOPPPS) teaching method to fit the characteristics and needs of oral histopathology. We then combined the characteristics of Small Private Online Courses (SPOCs) and a Flipped class to complete teaching material online, and assessed the effects of such teaching using a questionnaire and interviews. Fifty 5th-year undergraduates in stomatology at the School of Stomatology of Harbin Medical University of China participated in online classes. All were in the junior second half of the semester at the beginning of 2020. Teachers investigated from various medical colleges were responsible for delivering courses associated with stomatology or ophthalmology.

**Result & conclusion:**

The results showed that the modified BOPPPS combined with SPOC and the Flipped class improved teaching satisfaction. Modified BOPPPS combined with SPOC and the Flipped class is a useful complement to offline teaching on 5th-year undergraduate oral histopathology learning in China during COVID-19, and it can meet the multiple needs of students participating in the course.

**Supplementary Information:**

The online version contains supplementary material available at 10.1186/s12909-021-02980-6.

## Introduction

Students were prevented from going to campus with the outbreak of COVID-19 in early 2020. According to the requirements of the Ministry of Education in China, colleges and universities actively continued to deliver courses online and guaranteed teaching progress and quality during that period (http://www.moe.gov.cn/jyb_xwfb/gzdt_gzdt/s5987/202001/t20200127_416672.html). The Chinese government encouraged the use of online course platforms of all types at all levels and campus network learning methods to implement its “Suspension does not mean the cessation of teaching and learning” policy [1].

Compared with the face-to-face classroom, online teaching is not a mainstream teaching mode due to the absence of deeply interactive associations between students and teachers. However, under unavoidable pandemic status, the Internet had to become the source of learning and teaching. Hence, online teaching modes have become prevalent. In the long term, online teaching could be transformed to complement various other teaching methods.

Oral histopathology is a basic course in stomatology. The normal and pathological states of embryos and tissues can be determined by microscopy, which guides clinical treatment and provides prognostic information. It is the initial discipline for training advanced talents of stomatology, and also the bridge and cornerstone of clinical surgical skills and research into stomatology.

Here, we integrated Bridge-in, Objective, Pre-assessment, Participatory learning, Post-assessment and Summary (BOPPPS), the Small Private Online Course small-scale restrictive Online Course (SPOC), and the Flipped class for student-centered basic teaching online based on a communicative approach. The BOPPPS model adopts a modular decomposition method to cut the teaching process into multiple units of 5 to 15 min [2, 3]. The SPOC [4–6] and Flipped class [7, 8] are modules that are respectively embedded into multiple teaching units of the theory and practice of oral histopathology, and some experiences with this process are shared herein.

**Fig. 1:**
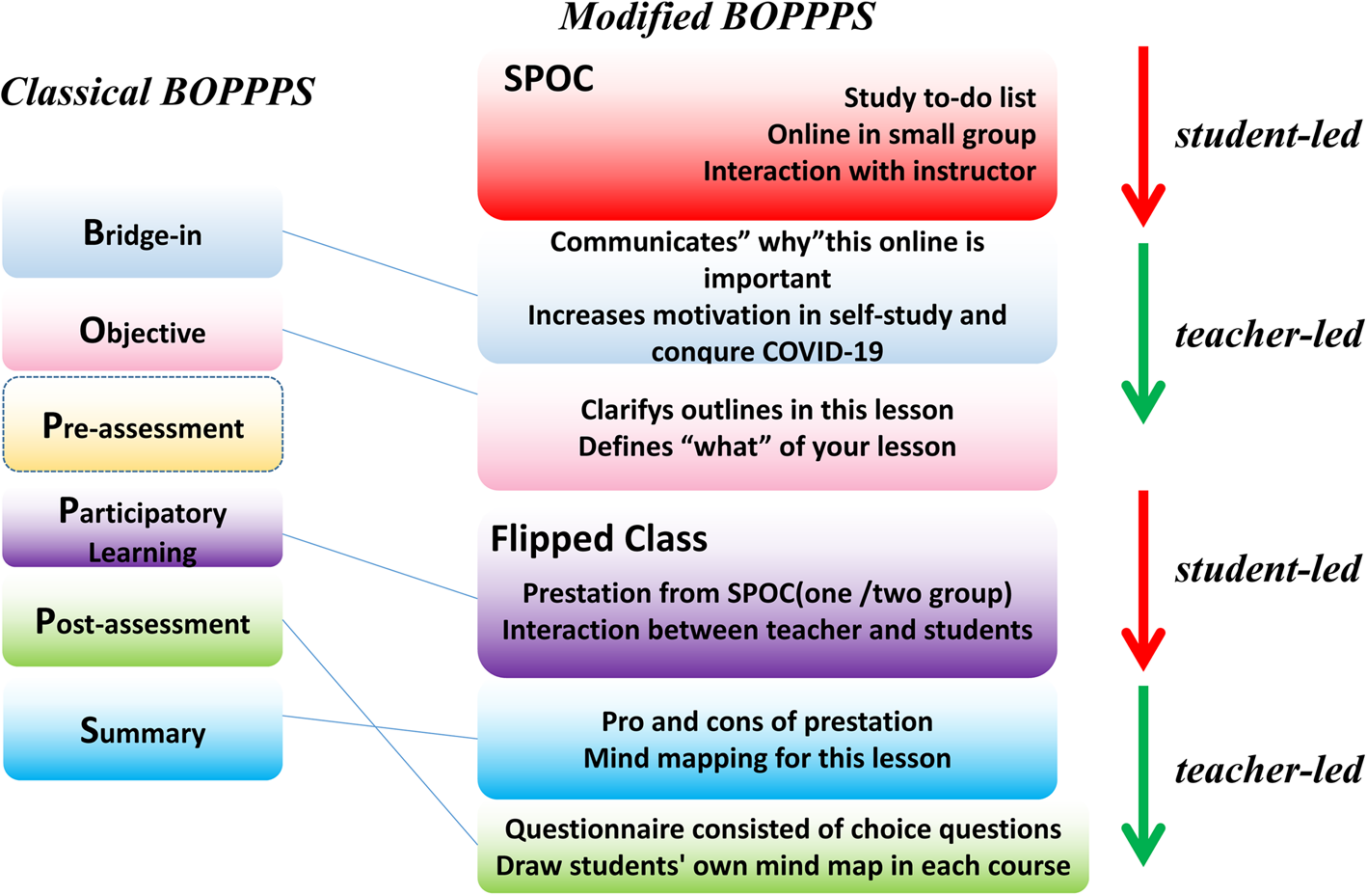
Flow of diagram on conceptual design based on BOPPPS

The teaching theory proposed by Douglas Kerrin in 1978 (BOPPPs) comprises six stages that form a systematic and complete teaching process with specific operational steps. To further focus on student participation, interaction, and feedback ability and strengthen the closed-loop teaching model, the original BOPPPS concept was modified into BOPPS. The specific changes were that a proportion of students learned a knowledge unit in advance under the guidance of the teacher through the SPOC model. The knowledge gained was then presented in an online class by a representative who described the students as a “Flip class.” This process replaced the pre-assessment in the original BOPPPS, and Fig. 1 shows the detailed process in the curriculum.

## Methods

### Implementation of modified BOPPS

#### Preparation by students and teachers

Fifty 5th-year undergraduates students participated in the online class. All were in the junior second-half semester at the School of Stomatology of Harbin Medical University of China. The participants were randomly assigned to nine study groups, and teachers arranged nine related knowledge units for each group and processed SPOC-learning, which were accompanied with a study to-do list (Supplementary Figure 1A), 2 weeks before the online class started. Teachers participated in the SPOC learning process for each study group. The knowledge units in SPOC were generally easy to understand or overlapped with previous learning content. Online resources for SPOC included the China MOOC APP and relevant reference books at home and elsewhere. Whole Slide Image online was offered instead of an actual microscope.

#### Framework and teaching content of modified BOPPPS

1. **Bridge-in (B):** Why this online method is important, and why it increases motivation for self-study in the face of the COVID-19 pandemic were communicated. Course content and ideological and political content in the China education system were introduced.
2. **Objectives (O):** Lesson outlines were drafted according to the syllabus of the stomatology major at Harbin Medical University and the syllabus of the Stomatology Practicing Physician Examination in China were clarified.
3. The teacher described the theoretical content and interacted with students through questions.
4. **Participatory learning (P):** The knowledge gained from SPOC by the Flipped Class was presented. The schedule of the Flipped class was assigned 2 weeks before the class. One or two groups were arranged to complete the Flipped class for each lesson. To ensure better effects, the groups watched a video from the China Massive Open Online Course (MOOC) platform and read reference materials that were associated with their presentation. A PowerPoint (PPT) presentation was delivered according to a standard set by teachers. The Flipped class generally consumed 16.6–20% of each course period and was arranged after a teacher delivered a presentation. Only one or two of the 9 groups underwent SPOC learning. The other groups learned this part of the teaching content based on new knowledge acquired in each course. During the flipping process, the teacher analyzed and highlighted errors in the presentation through direct interplay with all students.
5. **Summary (S):** The teacher provided a timely summary and positive feedback about the performance in each group, and corrected errors in the PPT explanation in the Flipped class. At the end of the lesson, the teacher organized all the knowledge threads of the lesson by mind mapping (Supplementary Figure 1B).
6. **Post-assessment (P):** Teachers assessed learning effects using a questionnaire comprising five multiple-choice questions (Supplementary Figure 1C). The teachers also encouraged students to draw their mind maps in each course (Supplementary Figure 2).

#### Evaluation of modified BOPPPS in the online teaching method

Quantitative data were acquired using questionnaires, and qualitative data were acquired by collecting feedback from students after class and conducting personal interviews.

## Results

### Perception regarding pre-assessment deletion

We found that 90% of the students had basic knowledge about medical morphology due to previous studies in histology, embryology, and pathology, which differs from the teaching systems in Europe and the USA. Although 50% of the students agreed that a pre-assessment is necessary for evaluating their status, 74% of them stated that they would be unwilling to involve themselves in a quiz, and 90% would feel anxious. Almost all classmates agreed to delete the Pre-assessment section. Table 1 shows the survey on the perception of deleting the Pre-assessment.

**Table 1:** Survey on the recognition of deleting ‘pre-assessment’-test

	Question		Frequency	Percent
1	Could you access your level of knowledge of histology, embryology and pathology?	A. Very well	30	60.00%
		B. Good	15	30.00%
		C. Average	5	10%
		D. Poor	0	0%
2	Do you think it is necessary to enter a quiz for evaluating student’s level in each course?	A. Necessary	5	10%
		B. No sense	25	50%
		C. Not necessary	20	40.00%
3	Do you be willing to enter a quiz for evaluating student’s level in each course?	A. Willing	0	0.00%
		B. No sense	13	16.00%
		C. Unwilling	37	74.00%
4	Do you be anxious to enter this quiz?	A. No sense	5	10.00%
		B. A little	30	60.00%
		C. So much	15	30.00%
5	Do you agree to delete the Pre-assessment’-test?	A. No sense	5	10.00%
		B. Totally agree	45	90.00%
		C. Disagree	0	0.00%

### Perception of SPOC by students

In the questionnaire regarding the perception of the SPOC, 28% of the students indicated that they liked the SPOC because of the active atmosphere with deep participation. Some students even stated that they felt like a teacher in a classroom in the personal interviews after class. Moreover, 28% also stated that SPOC is a good choice. In contrast, 9% of the students disliked SPOC because it was time consuming, coordination among the group members was reduced and the presentation was poor. Seven students wanted to have teachers deliver the SPOC presentation instead of the students. The frequency of SPOC implemented online showed that 26 (52%) participants felt that SPOC was organized, and that once every three lessons would be a good frequency. Thirteen (26%) participants preferred a longer interval, such as, “3–5 times per semester.” Six (12%) students responded positively to SPOC and they preferred a frequency of “once per lesson,” whereas 5 (10%) students had little interest in SPOC. Table 2 shows the survey regarding the perception of SPOC by the students.

**Table 2:** Survey on the recognition of “SPOC” from students

	Question			Frequency	Percent
1	Do you like SPOC?	A. Like it	a. Active initiative	4	8.00%
			b. Better sense of participation	5	10.00%
			c. Better sense of interaction	5	10.00%
		B. Not too bad		27	54.00%
		C. Dislike it	a. More time in this course	3	6.00%
			b. Difficulty in coordination with team members	4	12.00%
			c. Poor presentation capability in SPOC	2	4.00%
2	Do you hope to replace students’ SPOC presentation by teachers’?		A. No, students were excellent	8	16.00%
			B. No sense	7	14.00%
			C. Both students and teachers are OK.	28	56.00%
			D. Yes, students were not good at it .	7	14.00%
3	Which choice do you can accept the frequency of SPOC in the whole course.		A. Once each lesson	6	12.00%
			B. Once three lesson	26	52.00%
			C. 3-5 times in a semester	13	26.00%
			D. None in a semester	5	10.00%

### Perception of teachers regarding SPOC

We collected data from 15 teachers who shared their experiences with SPOC (Table 3). Topics included oral histopathology, oral maxillofacial surgery, oral care, and ophthalmology in different universities and all shared teaching. Most teachers like SPOC owing to the active initiative of the students and the performances were excellent. Nevertheless, one teacher noted that the preparation of SPOC resulted in spending more time and energy while instructing the students.

**Table 3:** Survey on the recognition of ‘SPOC’ from teachers

	Question			Frequency	Percent
1	Do you like SPOC?	A. Like it	a. Active initiative	14	93.33%
			b. Better sense of participation		NA
			c. Better sense of interaction		NA
		B. Not too bad			NA
		C. Dislike it	a. More time in this course	1	6.67%
			b. Difficulty in coordination with team members		NA
			c. Poor presentation capability in SPOC		NA
2	Do you hope to replace students’ SPOC presentation by teachers’?		A. No, students were excellent	13	86.67%
			B. No sense	1	6.67%
			C. Both students and teachers are OK.		NA
			D. Yes, students were not good at it.	1	6.67%
3	Which choice do you can accept the frequency of SPOC in the whole course.		A. Once each lesson	5	30.00%
			B. Once three lesson	5	30.00%
			C.3-5 times in a semester	4	26.67%
			D.None in a semester	1	6.67%

N*A* Not applicable

### Perception of teaching oral histopathology

Among the students, 70% thought that oral histopathology was interesting, 30% felt it was acceptable, and no one deemed the course boring (Table 4). The satisfaction rate of online teaching reached 100%, of which 62 and 38% were respectively, very satisfied and satisfied (Table 1). In the survey of how much teachers felt that they can compensate for face-to-face teaching, 18% thought online teaching can completely offset face-to-face schooling, whereas 37 (74%) of the 50 students considered that teaching online can compensate to a large extent and that Whole Slide Image can compensate for observation using a real microscope. Four students felt that online teaching could not compensate for face-to-face schooling or only to a limited extent.

**Table 4:** Survey on the recognition of the online teaching of oral histopathology

	Question		Frequency	Percent
1	Do you think oral histopathology is an interesting subject?	A. Interesting	35	70.00%
		B. Common	15	30.00%
		C.A little boring	0	0%
		D. Very boring	0	0%
2	Are you satisfied with the teaching effect of this online course?	A. Dissatisfied	0	0%
		B. Common	0	0%
		C. Satisfied	19	38.00%
		D. Well satisfied	31	62.00%
3	To what extent do you think online teaching can make up for the deficiency of face-to-face teaching?	A. < 40%	2	4.00%
		B. 40-60%	2	4.00%
		C. 60-80%	37	74.00%
		D. 80-100%	9	18.00%
4	Is it a pity that they did not observe the sections under a microscope?	A. Yes, microscope is necessary in my study	5	10.00%
		B. No sense	3	6.00%
		C. Whole slide image were enough	42	84.00%

## Discussion

The COVID-19 global pandemic has cause an unprecedented impact on global public health and affected the medical education sector, with many universities halting campus-based teaching and examinations [9–11]. The “Suspension does not mean the cessation of teaching and learning” policy was implemented in China at the start of 2020. We collected experiences with the online teaching model applied to oral histopathology early during the pandemic. We evaluated them according to current perspectives to benefit online and offline hybrid teaching reform in the future.

### Pros and cons of deleting re-assessment from BOPPPS

The original aim of the Pre-assessment in BOPPPS was to determine the general knowledge and capacity of students to participate in courses so that they could accurately adjust and appropriately deliver course content [12, 13]. We reformed the curriculum and removed the Pre-assessment based on the following factors. Firstly, our participants studied histology and embryology during the first semester of their first year according to the Syllabus of Harbin Medical University and had the basic capacity to understand morphological changes. Secondly, removing the Pre-assessment helped to reduce anxiety, particularly during the COVID-19 pandemic. The aim of online teaching during this period was to stimulate the interest of students to learn and transform fear associated with the pandemic using internal motivation. Table 1 shows that the Pre-assessment quiz could increase anxiety in the students. Deleting the Pre-assessment conferred an advantage during the early days of the pandemic. Subsequent investigations in other countries also found an obvious impact of COVID-19 on examination results and medical student placement, which decreased confidence and increased anxiety [9].

### Pros and cons of SPOC combined with the Flipped class

The aim of oral histopathology, as a professional basic course of stomatology, is to deepen learning ability. We encouraged the Flipped class [14, 15] to feel personally committed, and to ask for and provide relevant feedback from and to students and teachers in SPOCs to consequently promote deep learning [16, 17]. The data showed that one of the 50 students did not wish to involve SPOC in this process, although teachers provided consultation in time. Five of the 50 students liked the combination of SPOC and the Flipped class, as they felt very motivated during the interaction. We believe that the difference was mainly due to various types of learning motivation in each individual. Some students who believe that “passing is a good choice” can hinder the enthusiasm of students and the root of this might be a long-term, complicated process in basic Chinese education. Teachers also spent considerable time and energy to prepare SPOCs, which might be adjusted in the future. Elementary schoolteachers also responded that preparing materials suitable for the Flipped class was laborious and time-consuming under the effects of the COVID-19 pandemic [18].

However, the teachers believed that new thoughts and understanding through SPOC can be collected during the interaction of “teaching and learning.”

### Results of compensating for the absence of face-to-face instruction

The SPOC plus the Flipped class format used during the pandemic eased the transition from face-to-face teaching to online instruction [18–20]. Pre-class preparation resources and active learning materials that were already in place for flipped teaching were helpful in the transition to online teaching [20]. Therefore, the focus during the transition was to reconfigure active learning and examinations from the face-to-face format to the online platform by modified BOPPPS. According to the survey, 100% of the students were satisfied with online teaching and 70.4% of them who were particularly satisfied, thought that they could form a basic knowledge framework for oral diseases via oral histopathology lessons. Therefore, the main aim of this online lecture on oral histopathology was achieved under the unique circumstances imposed by the COVID-19 pandemic. About 10% of the students thought that being unable to observe sections under an actual microscope was unfortunate, but most believed that the digital sections could compensate to some extent. A few students liked the online teaching method very much and even suggested that online and offline mixed teaching should be the main form of future learning. This indicated that the individualization of teaching and learning is gradually becoming obvious. This small-scale survey during a global pandemic found that education administrators in China should focus not only on personalized education for the students, but also on providing a platform for the personalized development of teachers, especially young teachers.

## Conclusion

We believe that the modified BOPPPS combined with SPOC and a Flipped class, can greatly improve autonomous learning when students are denied access to a campus or face-to-face instruction on 5th-year undergraduate oral histopathology learning in China during COVID-19. We also found improvements in teachers during this process, which substantiates a Chinese proverb stating that teaching others teaches yourself. The integration and effective use of various network resources is helpful for further improving the knowledge system, and understanding existing knowledge. These experiences should be considered when considering online and offline hybrid teaching.

## Supplementary Information

**Additional file 1: Figure 1.** A. a study to-do list (in Chinese), which were assigned to students in advanced; B. a mind-mapping from teacher (in Chinese); C. a feedback from one quiz in the section of post-assessment from a lesson about jaw disease. **Figure 2.** This picture is a student’ mind-mapping of a lesson named benign salivary gland tumor.

## Data Availability

The data are kept at Department of Oral Pathology, Hospital of Stomatology, the First Affiliated Hospital, Harbin Medical University, Harbin 150001, P. R. China. Any questions or requests regarding the data can be addressed to Shan Wang (birchtree20032003@126.com).
