# Supplementary material for: Effects of modified BOPPPS-based SPOC and Flipped class on 5th-year undergraduate oral histopathology learning in China during COVID-19
Source: BMC Med Educ. 2021 Oct 26;21:540. doi: 10.1186/s12909-021-02980-6 (PMC8546376; doi:10.1186/s12909-021-02980-6)
Supplement: Supplementary file 1 — Additional file 1: Figure 1. A. a study to-do list (in Chinese), which were assigned to students in advanced; B. a mind-mapping from teacher (in Chinese); C. a feedback from one quiz in the section of post-assessment from a lesson about jaw disease. Figure 2. This picture is a student’ mind-mapping of a lesson named benign salivary gland tumor. [file 12909_2021_2980_MOESM1_ESM.docx]

**C**


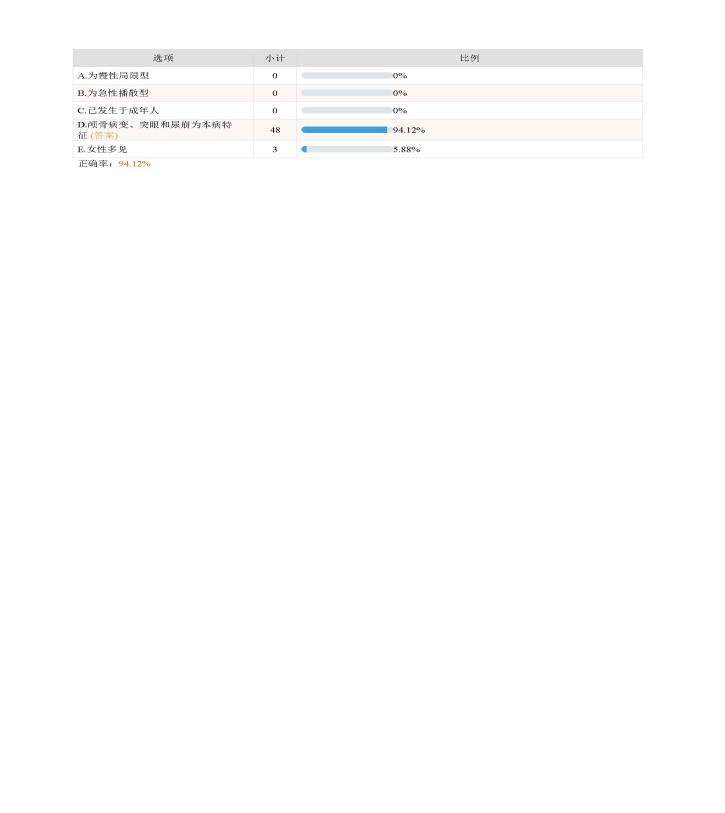

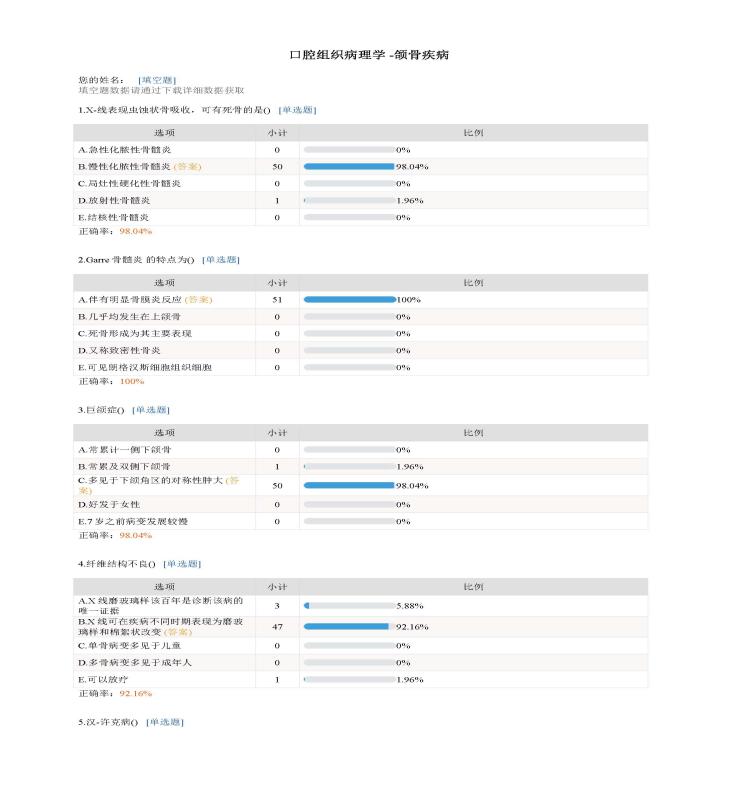


**A**


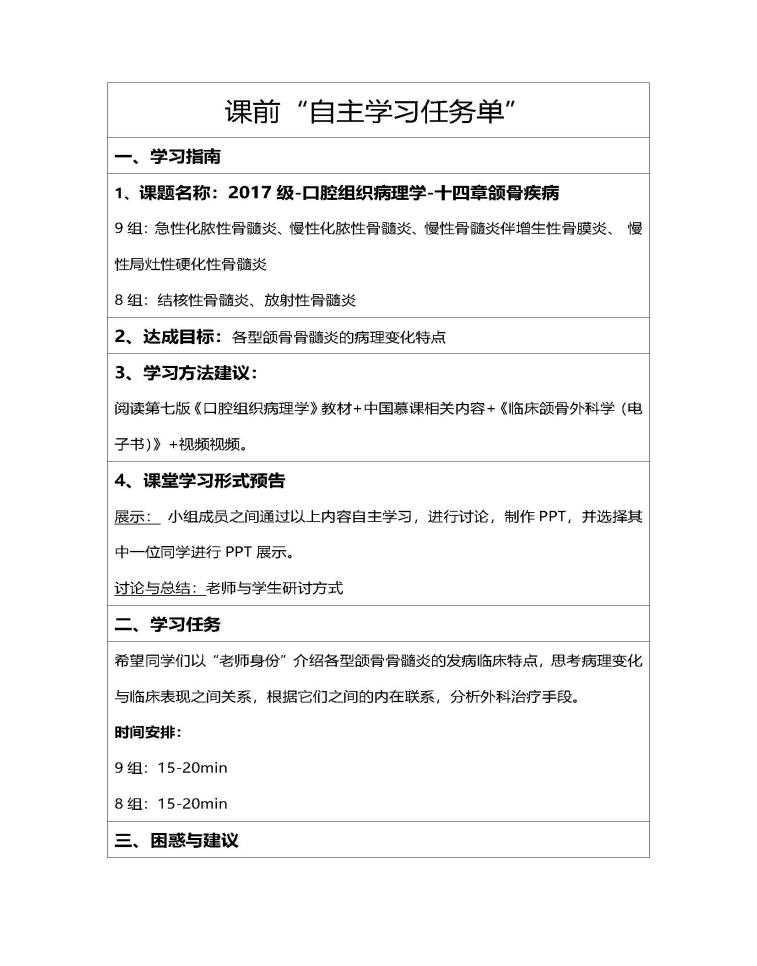


**B**


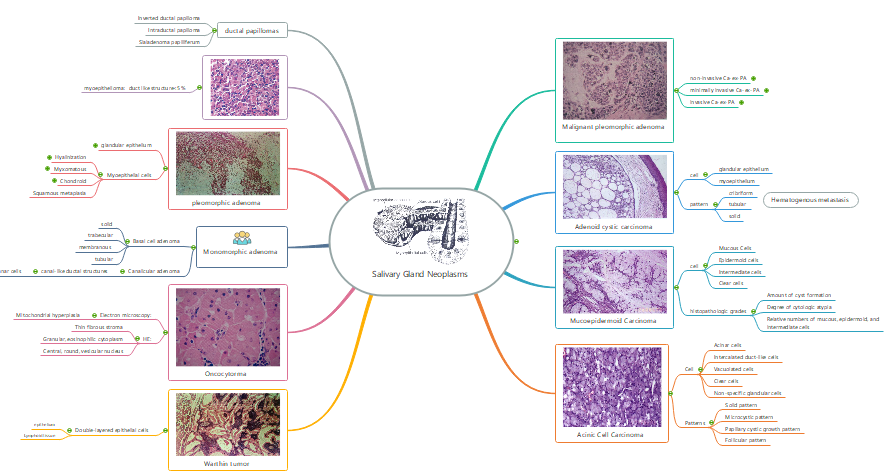


**Figure.1 A. a study to-do list(in Chinese), which were assigned to students in advanced; B. a mind-mapping from teacher(in Chinese); C. a feedback from one quiz in the section of post-assessment from a lesson about jaw disease.**


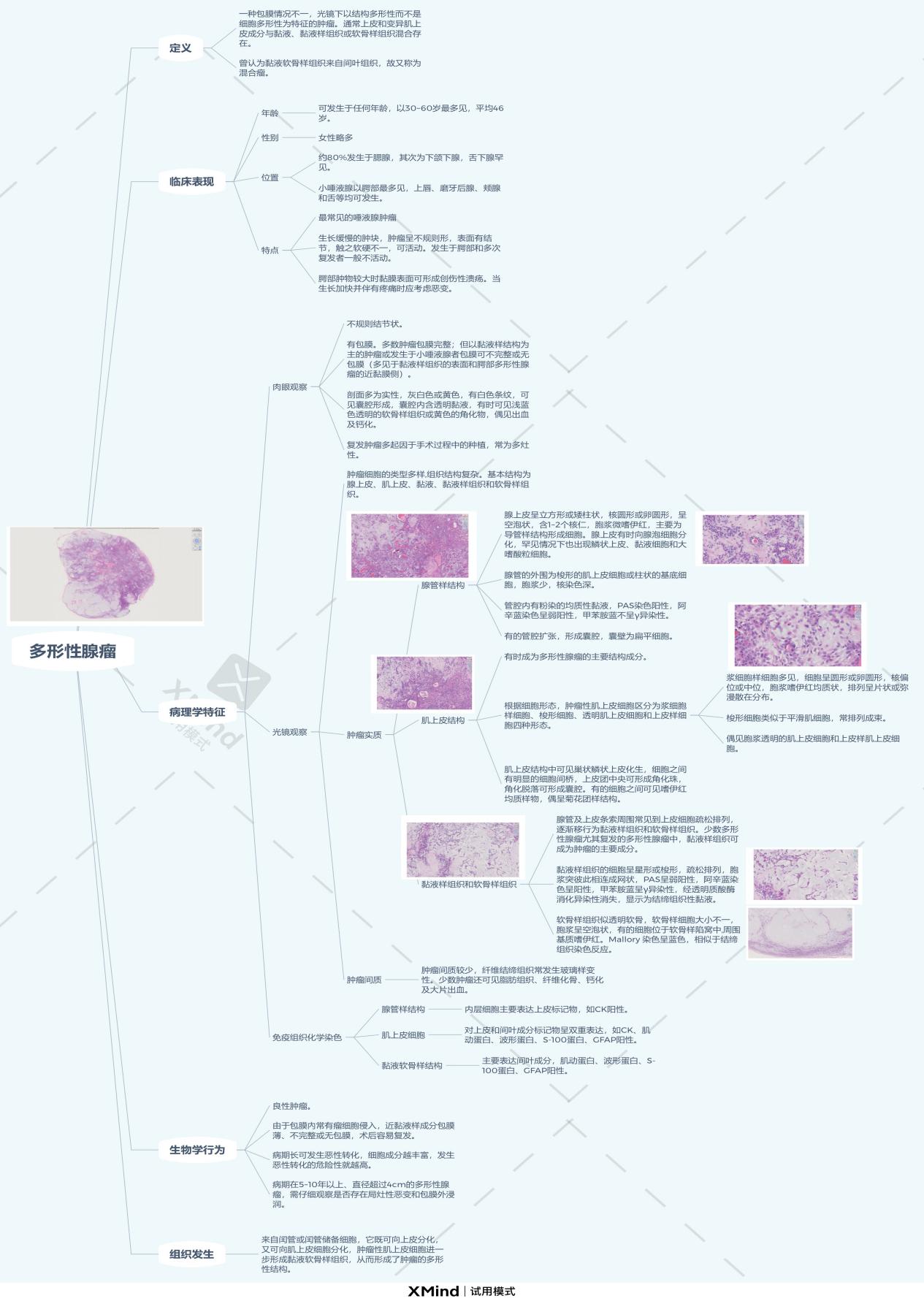


**Figure.2. This picture is a student’ mind-mapping of a lesson named benign salivary gland tumor**
